# The appropriateness of penicillin allergy de-labelling by non-allergist clinical ward teams

**DOI:** 10.1016/j.clinme.2024.100225

**Published:** 2024-06-27

**Authors:** Neil Powell, Shuayb Elkhalifa, Daniel Hearsey, Michael Wilcock, Jonathan Sandoe

**Affiliations:** a Pharmacy Department, Royal Cornwall Hospital Trust, Truro, UK; b Allergy and Immunology Department, Respiratory Institute, Cleveland Clinic Abu Dhabi, Abu Dhabi, United Arab Emirates; c Leeds Institute of Medical Research, University of Leeds, Leeds, UK and Leeds Teaching Hospitals NHS Trust, UK

**Keywords:** Non-allergist, Penicillin allergy, Penicillin allergy de-labelling

## Abstract

**Objectives:**

We aimed to assess the appropriateness of penicillin allergy (PenA) assessment conducted by clinical teams and to review the safety of subsequent exposure of these patients to penicillin.

**Methods:**

Opportunistic, prospective observational study of usual clinical care, between 16 May 2023 and 14 August 2023, of inpatients with a PenA and requiring antibiotics, in a 750-bed hospital in England. To assess the appropriateness of management, PenA patients prescribed penicillins were grouped into risk categories using a validated antibiotic allergy assessment tool: eligible for de-label on history alone (direct de-label; DDL), eligible for direct oral challenge (DOC), high risk or unable to obtain history.

**Results:**

Of the 123 patients admitted with a PenA (or sensitivity record) and exposed to a penicillin, data were collected for 50. Their PenA records were grouped follows: eligible for DDL 34 (68%), eligible for DOC 11 (22%), high risk 4 (8%) and unable to obtain history 1 (2%). In 14/50 (28%) patients there was no evidence of a current PenA assessment in the medical notes.

**Conclusions:**

Using the allergy risk tool, most patients with PenA records were exposed to penicillin appropriately. However, patients meeting high-risk criteria were also exposed to penicillin when the tool excluded them. PenA assessment needs to be carried out with appropriate training and governance structures in place.

**Summary box**

**Table:** 

What is known?	Penicillin allergy records are common and often incorrect. They prevent patients receiving first-line antimicrobial therapy which has know negative consequences. In our hospital, ward clinical teams are intentionally exposing patients with penicillin allergy records to penicillin so that they receive first-line antimicrobial therapy.

What is the question?	To review the safety of penicillin allergy de-labelling delivered by the ward-based clinical teams.

What was found?	The majority of patients with PenA records and exposed to penicillin had a low-risk allergy history appropriate for re-exposure to penicillin. A small proportion had PenA histories that we would categorise as high risk. There was no reported harm in any of the patients challenged with penicillin.

What is the implication for practice now?	As part of ongoing work in the hospital to safely remove incorrect PenA records, the next steps are to publish a local PADL guideline and offer ward doctors, nurses and pharmacists training in safely removing incorrect penicillin allergy records.

## Introduction

Penicillin allergy (PenA) labels are common and associated with negative patient and health system outcomes.^1^ There are many examples of non-allergy specialist healthcare workers tackling incorrect PenA labels as part of formal de-labelling programmes; the majority of these involve PenA de-labelling (PADL) interventions delivered by healthcare workers working in antimicrobial stewardship (AMS) roles.^2^ Details of PADL, carried out during the course of usual clinical care by the teams responsible for inpatients, are rarely reported.^2^

We piloted a 3-month, pharmacist-led, multidisciplinary penicillin allergy de-labelling daily ward round to determine the opportunity for penicillin allergy de-labelling in a UK hospital starting in November 2022. During the pilot, we observed patients with PenA records being prescribed penicillin by their responsible clinical teams. This prescribing happened outside our AMS-driven PADL study and in the absence of specific PenA de-labelling guidelines in our hospital.^3^

### Study aims

1. To assess the appropriateness of the PenA risk assessment conducted by ward-based clinical teams.
2. To review the safety of subsequent exposure of these patients to penicillin.

## Methods

### Ethics

This service improvement project did not require ethics approval.

### Study design

Opportunistic, prospective, observational, descriptive study of usual clinical care of patients with a PenA requiring antibiotic therapy.

### Setting

A 750-bed hospital in England, serving a population of 430,000 people, and without allergy services.

### Inclusion criteria

A weekly ward round identified inpatients on any medical ward between 16 May 2023 and 14 August 2023 with either a current PenA (or sensitivity) record or a PenA record removed during the current inpatient stay, and also prescribed penicillin.

### Variables collected

The following data were collected for included patients: admission date, age, clinical specialty responsible for the penicillin prescription, PenA history as documented in the electronic prescribing and medicines administration (EPMA) drug allergy section, sensitivity history as documented in the EPMA sensitivity section, PenA history as documented in the GP records, PenA history if taken from medical/pharmacy teams and documented, PenA history taken from patient by AMS pharmacists (if not documented by the responsible clinical teams), penicillin prescribed and indication, National Early Warning Score (NEWS) at time of first penicillin dose, PenA risk category, discharge date, and any documented evidence of harm from the penicillin.

### Data sources

A live web feed generated by the pharmacy data analysis team identified inpatients with a PenA record (either showing on the inpatient EPMA record or removed from their EPMA record during the current episode of care) or a penicillin sensitivity record currently showing in EPMA, and prescribed a penicillin during their current admission. The EPMA system has two places where adverse drug reactions can be recorded: an allergy section and a sensitivity section. The allergy section has built-in decision support so that if a documented allergen is prescribed, the system alerts the prescriber and mandates a reason for prescribing the known ‘allergen’. Removal of information in the allergy section is recorded within the EPMA system and leaves an auditable trail of changes. The sensitivity box has neither an alert function nor an audit trail.

Using the documented PenA or sensitivity history, if obtained during the current episode of care or, if missing, taken by the auditor (AMS pharmacist), patients were grouped into the following categories using the decision support tool developed by Devchand *et al*:^4^ eligible for de-label on history alone (direct de-label; DDL), eligible for direct oral challenge (DOC), high risk or unable to obtain history. Descriptive statistics were used.

## Results

**Fig. 1:**
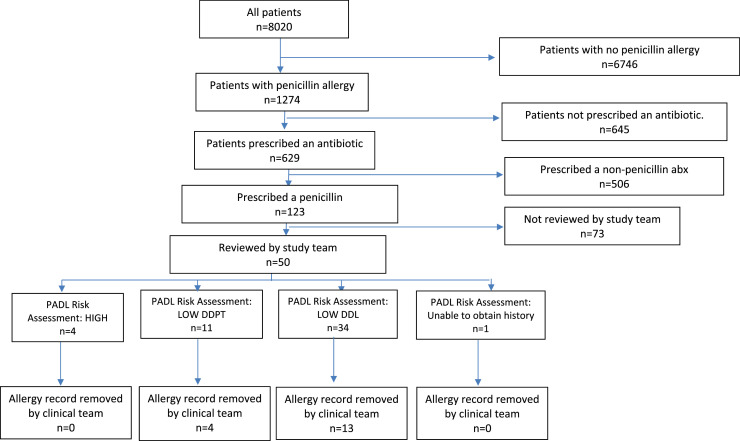
Study flowchart.

During the study period, 8,020 patients were discharged who had spent some, or all, of their stay on an included medical ward, of whom 1,274 (15.89%) had a PenA or sensitivity record on admission. Of these 1,274 patients, 629 (49.37%) received an antibiotic, of whom 123 (19.55%) received a penicillin. The 123 who received a penicillin comprised 95 with a PenA record and 28 with a penicillin sensitivity (Fig. 1 & Table 1).

**Table 1:** Patient demographics – summary.

Characteristic	Patients (*n*=50)
Sex Male Female	22 (44%) 28 (56%)
Age (years) Median IQR	78 19 (66–85)
Documented Allergy Sensitivity	35 (70%) 15 (30%)
Specialities responsible for care of patient when de-labelled Acute medicine Emergency department Respiratory Endocrine Infectious diseases General surgery Gastrointestinal Cardiology Unknown	15 10 9 4 4 3 1 1 1
Name of index penicillin recorded in PenA record Penicillin(s) Flucloxacillin Amoxicillin Phenoxymethylpenicillin Co-amoxiclav Piperacillin/tazobactam Unknown	14 11 10 10 1 1 2
Penicillin prescribed Piperacillin/tazobactam Amoxicillin Flucloxacillin Benzylpenicillin	21 18 13 1
Infections treated (by organ) Respiratory Skin, skin structure and bone Gastrointestinal Neutropenic sepsis or sepsis, unknown source Cardiac Renal tract Bacteraemia	17 13 7 5 3 2 2
DOC allergy histories Unknown reaction >10 years ago Childhood rash Isolated swelling Unknown childhood reaction Rash >10 years ago DDL allergy histories GI upset Subsequent tolerance of penicillin since index reaction Patient denies PenA Dizziness Generally unwell Felt panicky Drowsiness Penicillin record added to EPMA system in error High-risk allergy histories Shortness of breath Widespread urticarial rash Mouth ulcers Blistering rash Unable to obtain history	11 (22%) 5 2 2 1 1 34 (68%) 13 11 4 2 1 1 1 1 4 (8%) 1 1 1 1 1 (2%)

Of the 95 patients with a PenA who subsequently received penicillin, 55 (58%) had their PenA record removed by the clinical teams and 40 (42%) did not. Amendments to sensitivity records are not audited within the EPMA system, and so determining whether the record was amended was not possible, but they were not removed, which may or may not have been appropriate

We retrospectively reviewed (ie after the penicillin prescription had taken place) the management of 50 of these individuals (a sample of convenience) during the weekly ward rounds (Table 1). For 31/50 (62%) patients there was evidence that a PenA history had been taken during the episode of care, by either the ward medical or ward pharmacy staff. In 14/50 (28%) patients there was no documented evidence of a current PenA assessment having taken place, and in 5/50 (10%) cases the medical notes referred to the EPMA PenA section, so it was unclear if the PenA history has been taken from the patient during current episode of care. According to the PenA history, the 50 patients were retrospectively grouped as follows: eligible for DDL 34 (68%), eligible for DOC 11 (22%), higher risk 4 (8%) and unable to obtain history 1 (2%).

Of the 11 patients who had a PenA history categorised as eligible for DOC, there was evidence of a formal DOC documented in the notes with post-dose observations in three patients, and evidence of a DOC without mention of observations in one further patient. The remaining seven patients had been started on penicillin therapy without documentation of formal testing.

None of the patients categorised as a higher-risk PenA history had a documented allergy history or formal allergy assessment in the medical notes (Table 1). The patient with a history of ‘shortness of breath’ had penicillamine recorded in the allergy section on EPMA and received three doses of oral amoxicillin during the admission. The patient with a history of blistering skin rash received a stat dose of piperacillin/tazobactam before switching to doxycycline. The patient with a history of widespread urticarial rash received 5 days of piperacillin/tazobactam. The patient with a history of mouth ulcers who had since tolerated flucloxacillin from their GP, received piperacillin/tazobactam then amoxicillin to complete a 6-day course of antibiotics for COPD exacerbation. Except for the patient who had a documented reaction to penicillamine, the rest had penicillin in the EPMA PenA section and would have had a PenA alert from the EPMA system when prescribing a penicillin; this must have been overridden by the prescriber, ie prescribing of penicillin was intentional, not accidental. There was no evidence of patient harm documented in the medical notes because of exposure to penicillin in any of these high-risk patients.

Thirty-three of 34 patients in the DDL category were exposed to penicillin and had a median NEWS score 1 (IQR 0–3). Of the 11 patients in the DOC category and exposed to penicillin, their median NEWS score was 1 (IQR 1–3), and the four high-risk patients’ NEWS scores were median 6.6 (IQR 4–10).

Of 45 patients de-labelled by DOC/DDL; nine had a note in the discharge letter alerting the GP to the de-label, nine had died while an inpatient, making mention of de-label irrelevant, and the remaining 27 had no communication about their de-label.

## Discussion

Our study revealed that clinician-led PenA assessments and de-labelling were being carried out by our ward teams, despite the lack of official hospital guidelines and a governance structure. About 20% of patients with a PenA or sensitivity record, mainly those with low-risk histories, were appropriately prescribed penicillin.

We identified several areas for improvement. Documentation of a current PenA assessment was missing in the medical notes for a quarter of patients with a PenA and exposed to penicillin, of whom four had higher-risk allergy histories. Several ‘high-risk’ patients were given penicillin without allergy specialist input. Many patients who should have received a supervised DOC were given penicillin without documentation of formal oral challenge test or post-challenge dose observations. PenA record maintenance was poor with hospital EPMA systems inconsistently updated, and a minority of patients had evidence of GP communication about the patient's new allergy status. Ensuring that the hospital and GP records are updated to reflect the patient's new allergy status, as well as ensuring that patients are counselled on the benefits of PenA removal, are important to ensure the patient isn't re-labelled.

The allergy assessment tool, developed by Devchand *et al,* identified four patients as higher risk who should therefore have been excluded from penicillin exposure.^4^ The use of penicillin in these patients was outside the guidance by Devchand *et al;*^4^ however, other allergy assessment scoring tools may not have excluded these patients, depending on remoteness (time between index reaction and penicillin re-challenge) and severity of index reaction.^5^^,^^6^ There was no evidence of patient harm in the medical notes for any of the patients exposed to penicillin.

### Limitations

This was a review of prescribing in a convenience sample of patients in a single centre over a 3-month period. As it was a retrospective review, we couldn't ascertain the extent of patient counselling or consent that preceded the penicillin prescriptions. Due to resource constraints, we were limited to a once-weekly ward round and as such were able to only capture 50 of the 123 patients with a PenA who were exposed to a penicillin. We were not able to identify patients with amendments made to information in the sensitivity section of EPMA because there is no log of amendments to the sensitivity section recorded in EPMA, unlike the PenA section. As such, any patients admitted with a penicillin sensitivity entry that was subsequently removed while an inpatient and prescribed penicillin would be missed by our method of surveillance.

### Implications for practice and research

With only one-fifth of patients with a PenA exposed to penicillin, there is opportunity to increase the proportion to nearer 50%, as done by others.^2^ How to PADL in the hospital requires further study through qualitative interviews with patients and healthcare workers delivering PADL. We will publish local hospital PADL guidelines with education and competency-based training to ensure the safe delivery of PADL.

## Conclusion

Ward-based medical and pharmacy teams are undertaking PenA assessment and de-label as part of routine ward patient care. There is some evidence of formal PenA testing and some evidence of communication about new PenA status with primary care, but action is needed to ensure that PADL is safe and optimised in our study hospital, with appropriate training and governance structures in place.

## Declaration of competing interest

NP & JS have NIHR funding to explore how to best deliver penicillin allergy de-labelling. The work presented here was undertaken as part of usual work and is not funded.

## References

[1] Krah NM, Jones TW, Lake J, Hersh AL. (2021). The impact of antibiotic allergy labels on antibiotic exposure, clinical outcomes, and healthcare costs: a systematic review. Infect Control Hosp Epidemiol.

[2] Powell N, Stephens J, Kohl D (2023). The effectiveness of interventions that support penicillin allergy assessment and delabeling of adult and pediatric patients by nonallergy specialists: a systematic review and meta-analysis. Int J Infect Dis.

[3] Hearsey D, Elkhalifa S, Sandoe J (2023). Removal of incorrect penicillin allergy labels in a UK hospital. Clin Microbiol Infect.

[4] Devchand M, Urbancic KF, Khumra S (2019). Pathways to improved antibiotic allergy and antimicrobial stewardship practice: the validation of a beta-lactam antibiotic allergy assessment tool. J Allerg Clin Immunol Practice.

[5] Wiersinga WJ, Bonten MJ, Boersma WG (2012). SWAB/NVALT (dutch working party on antibiotic policy and dutch association of chest physicians) guidelines on the management of community-acquired pneumonia in adults. Netherlands J Med.

[6] Trubiano J, Chua K, Holmes N (2020). PEN-FAST: a validated penicillin allergy clinical decision rule - Implications for prescribing. Int J Infect Dis.

